# Thymoma-associated Myasthenia Gravis in a Young Adult with Development of Paraneoplastic Limbic Encephalitis and Systemic Lupus Erythematosus Post-thymectomy: A Case Report

**DOI:** 10.7759/cureus.3581

**Published:** 2018-11-13

**Authors:** Robin Liu, A. Rashid Dar, Keng Yeow Tay, Mike W Nicolle, Richard I Inculet

**Affiliations:** 1 Internal Medicine, Western University, London, CAN; 2 Oncology, Schulich School of Medicine & Dentistry, Western University, London, CAN; 3 Radiology, London Health Sciences Centre, London, CAN; 4 Neurology, University Hospital, London Health Sciences Centre, London, CAN; 5 Cardiac/thoracic/vascular Surgery, London Health Sciences Centre, London, CAN

**Keywords:** thymoma, myasthenia gravis, limbic encephalitis, paraneoplastic, autoimmune, thymectomy, young adult

## Abstract

We report a young adult with thymoma-associated myasthenia gravis (MG) who, following thymectomy, developed paraneoplastic limbic encephalitis (LE) and systemic lupus erythematosus (SLE). Although thymomas commonly co-occur with MG, LE is an uncommon autoimmune sequela. Herein, we discuss the pathophysiology of paraneoplastic LE and its management. Our report also highlights an unusual case of a thymoma patient who presented with multiple autoimmune disorders. The treatment of such a patient is therefore challenging and requires care from multiple specialized teams.

## Introduction

Thymomas are rare epithelial cell tumours arising from the thymus gland in the anterior mediastinum [1]. Patients with thymomas are often diagnosed incidentally but can present with dyspnea, chest pain and rarely superior vena cava syndrome from mass effects [1]. Importantly, many patients present with a variety of possible autoimmune manifestations, which prompts investigations for a thymic mass [2]. The management of these patients is complex and often involves the treatment of concurrent autoimmune disorders in addition to the underlying malignancy. In this case report, we discuss a 19-year-old patient with a thymoma who presented with myasthenia gravis (MG). She underwent surgery, chemotherapy and radiation and remained disease-free after 13 years of follow-up. Yet, following surgical resection, she developed limbic encephalitis (LE) that was seropositive for voltage-gated potassium channel autoantibodies (VGKC). She also continued to develop systemic lupus erythematosus (SLE) during follow-up.

## Case presentation

**Figure 1:**
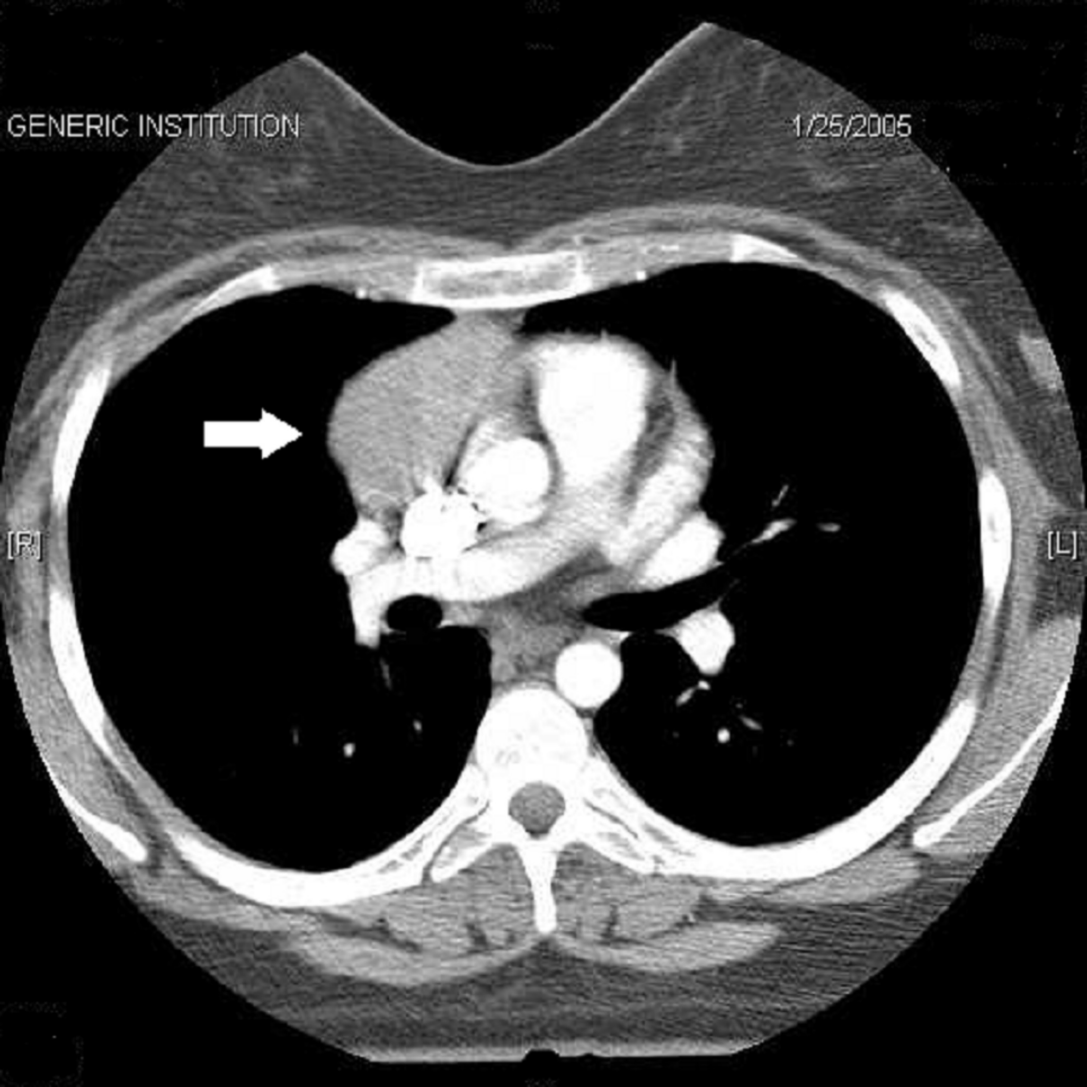
Right anterior mediastinal mass that extends along the pericardium from the level of the aortic arch to the right ventricle

A 19 year-old Caucasian woman presented to the neuromuscular clinic in November 2004 with generalized weakness, left-sided ptosis, fatigue and dyspnea at rest. She was diagnosed with anti-acetylcholine receptor antibody-positive MG and was treated with azathioprine (150 mg), prednisone (60 mg daily) and pyridostigmine (60 mg QID). An associated thymic abnormality was explored, and computed tomography (CT) of the thorax revealed a 4.3 x 5.7-cm (transverse x anteroposterior [AP]) anterior mediastinal mass, suspicious of a thymoma (Figure 1). Prior to surgery, her prednisone dose was tapered to 25 mg daily to reduce the likelihood of wound complications, and she underwent three courses of plasmapheresis (5-L exchanges) to reduce the chances of peri-operative worsening in her MG. A trans-sternal thymectomy was performed in April 2005 without complication. The pathology confirmed the diagnosis of a 107-g, 6.5 x 8.5 x 3.5-cm WHO (World Health Organization) type B2 thymoma that was microscopically invasive into the perithymic adipose tissue. No gross invasion was visible, and an octreotide scan revealed no distant metastases. Due to the presence of a thymomatous microscopic invasion, chemoradiation was planned to reduce the chances of local recurrence or intrapleural or systemic metastases.

**Figure 2:**
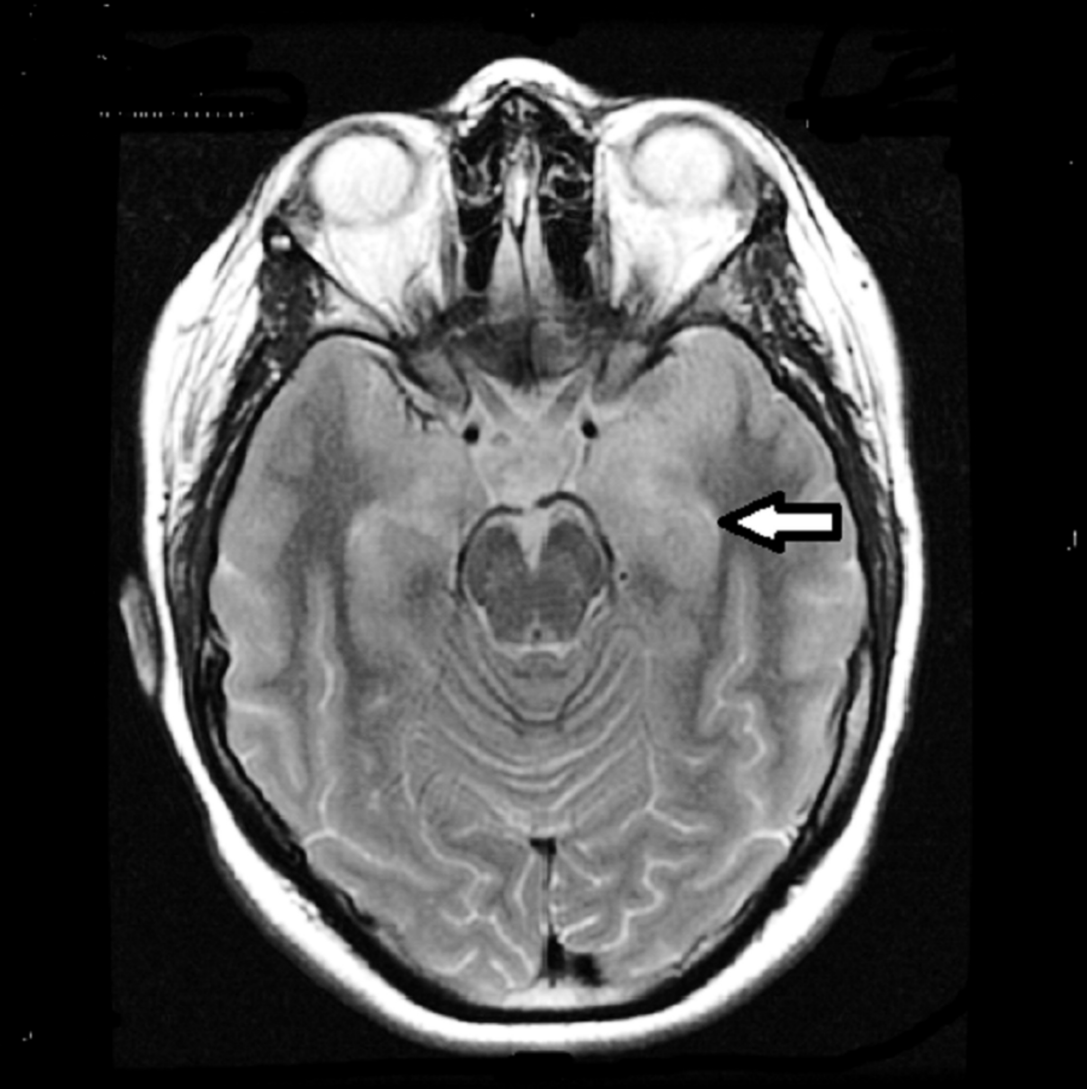
T2 hyperintensity in the anteromedial left temporal pole most prominently There is evidence of a mass effect with thickening of the cortex and effacement of the adjacent sulci

Plans for chemoradiation were delayed after she presented in May 2005 with episodes of altered awareness and post-ictal confusion. Following thymectomy, she had developed memory loss, receptive aphasia, confusion, lip smacking, delusions and visual hallucinations. Her electroencephalogram (EEG) showed bilateral temporal epileptiform activity, and a CT head showed a subtle loss of gray and white differentiation in the medial aspect of the left temporal lobe. She was admitted to the epilepsy unit and was investigated for herpes encephalitis and LE as the cause for her epilepsy. Magnetic resonance (MR) imaging scan of the head revealed effacement of the left insular cortex and hippocampus posteriorly and T2 hyperintensity in the left temporal lobe (Figure 2). Polymerase chain reaction (PCR) testing of the cerebrospinal fluid for the detection of herpes simplex virus (HSV) was negative, but her serum anti-voltage-gated potassium channel antibody (VGKC) was found to be elevated. During the course of her hospital stay (one month), she was started on valproic acid (750 mg BID) and topiramate (200 mg BID) and received a course of intravenous immunoglobulin (IVIg; 2 mg/kg given over three days) for the presumptive diagnosis of immune-mediated epilepsy.

One month later, she underwent three courses of doxorubicin and cisplatin given one month apart. Following chemotherapy, she received 4000 CGy of radiation to the anterior mediastinum in 22 fractions through a conformal technique to avoid damage to the normal tissue. She tolerated her radiation treatment well and did not develop complications to the treatment. As of her most recent CT thorax (July 2016), there was no evidence for a residual thymic tissue or a recurrent thymoma.

Her seizures were well controlled and she was able to discontinue the valproic acid but remained on topiramate (200 mg/day). After her thymectomy and with the medical treatment of her MG, she did well and the doses of her prednisone and pyridostigmine were tapered and discontinued in March 2006. However, in August 2010, she presented with a relapse of MG, which was treated by re-starting 120 mg QID of pyridostigmine and 20 mg of prednisone daily.

In May 2006, she developed polyarthralgias, oral ulcers, patchy alopecia and livedo reticularis on the hands and legs. Her history of thymoma and VGKC immune-mediated LE prompted a workup for autoimmune disease. Bloodwork revealed a positive antinuclear antibodies (ANA) test (1:320), normal complement, a negative rheumatoid factor, a positive extractable nuclear antigen (ENA) test and a double-stranded deoxyribonucleic acid (dsDNA) of 367 IU/mL (reference is less than 15 IU/mL). She was initiated on indomethacin (50 mg/day), hydroxychloroquine (400 m/day) and long-acting nifedipine (Adalat XL 30 mg/day). Her SLE is well-managed on medical treatment.

## Discussion

Our case highlights the complexity in the management of thymoma patients. In addition to the appropriate treatment of the underlying malignancy, the autoimmune sequelae that often present concurrently or post-operatively must also be managed. Oncologically, the treatment of thymomas generally involves surgical resection accompanied by adjuvant or neoadjuvant chemoradiation when there is evidence for microscopic or gross invasion [3-4]. Complete resection of the mass by thymectomy is the gold standard in the treatment of an early-stage thymoma and offers patients the best prognosis [5-7]. The National Comprehensive Cancer Network provides guidelines for management based on the tumour stage [5,8]. Chemoradiation is often used as a neoadjuvant therapy to permit the resection of previously unresectable masses [8]. It is also used as an adjuvant therapy in metastatic diseases. A cisplatin, doxorubicin and cyclophosphamide-based therapy, with or without prednisone, is recommended for the treatment of thymomas [8]. Radiation therapy can be used alone in an R1 resection, where there is only evidence of a microscopic tumour residue, but additional chemotherapy is recommended after an R2 resection, where there is a macroscopic residual tumour [9]. Despite aggressive therapy, advanced stage disease usually results in very poor prognoses [6-7].

In over half of the cases, the diagnosis of a thymoma is made concurrently with the presentation of an autoimmune disorder [2]. The most common autoimmune manifestation to occur is MG (present in 30-50% of thymoma patients); however, there is an extensive list of other rare disorders (LE, neuromyotonia, pure red cell aplasia, etc.) [2,10]. The strong association between thymomas and autoimmune disorders has been proposed to be the result of a disruption in the process of negative selection of autoreactive T cells by the tumour microenvironment [10]. A recent gene expression study comparing the thymoma tissues from MG-positive patients and MG-negative patients provides some evidence supporting the role of thymoma tissues in MG pathogenesis [11].

In addition to MG, our patient developed a second rare autoimmune disorder post-thymectomy: LE with serum positive for anti-VGKC autoantibodies. The classic presentation of the LE is acute memory loss with psychiatric manifestations and often seizures [12]. Paraneoplastic LE is more commonly associated with lung, breast, ovarian and uterine cancers, while few thymoma patients have been reported with this autoimmune manifestation [2,13].

Autoimmune LE is associated with the presence of autoantibodies that target the neuronal antigens [13]. The disorder’s association with certain malignancies, its response to treatment and the presenting clinical picture are related to the specific offending autoantibody [13]. These autoantibodies are firstly categorized as either onconeuronal, targeting epitopes within the neurons, or cell-surface autoantibodies that target epitopes on the surface of neurons [13]. Autoimmune LE with onconeuronal autoantibodies, like anti-Hu antibodies commonly found in small cell lung cancers, do not respond well to immunotherapy or cancer treatment compared to LE due to cell-surface antigens. The exception to this rule is LE associated with anti-Ma2 autoantibodies, which is responsive to immunosuppression [14].

In VGKC-associated LE, the autoantibody target is a cell-surface epitope and is responsive to immunotherapy and cancer treatment [15]. The VGKC-associated neurological diseases can be further broken down into the protein subunits they target [15]. For example, VGKC autoantibodies were first associated with neuromytonia, a disease characterized by peripheral nerve excitability [15]. However, in contrast to the autoantibodies found in VGKC-associated LE which immunoprecipitated the Kv1.1 subunit, those found in neuromytonia precipitate the Kv1.2 and Kv1.6 subunits [15]. Specifically, the pathogenic autoantibodies in VGKC-associated LE target LGI1 or CASPR2 [15]. Indeed, the reduction of VGKC titers is well-correlated with the clinical course after treatment, implicating these autoantibodies in disease pathogenesis [14].

While there are reported cases of patients improving with anticonvulsant treatment alone, most patients require an immunosuppressive treatment for VGKC-associated LE [16]. One study proposed that anticonvulsant therapy may lower the blood-brain-barrier permeability to the offending autoantibodies [16]. However, the mainstay of treatment for LE involves steroids, plasma exchange and IVIg [14]. Anticonvulsant and antipsychotic agents are used for symptom control only [14]. Recent studies have shown that starting with a combination of corticosteroids and IVIg can be beneficial to cognitive improvement but is associated with a higher incidence of iatrogenic complications [17]. Importantly, the early treatment of LE is correlated with a better clinical course as patients manifesting with symptoms for months prior to treatment do not recover completely [14]. If the patient is refractory to first-line treatment, plasmapheresis can be added [14]. Certain steroid-sparing agents such as rituximab have been used, but the reports are scarce in the literature and the results are variable [17].

The development of autoimmune conditions post-thymectomy is uncommon, as they tend to present concurrently or prior to thymoma diagnosis. Yet, our patient proceeded to develop both paraneoplastic LE and SLE post-resection. In Bernard et al.'s review of 85 thymoma cases, only six had presented with two or more autoimmune disorders and seven presented with autoimmune disease post-thymectomy [2]. Unfortunately, no risk factors for the development of autoimmune disorders post-thymectomy have been identified. 

## Conclusions

Interestingly, in our case, the patient presented with paraneoplastic LE post-resection. While the development of the autoimmune disorders could be independent of the thymoma, the association of thymoma with autoimmune disorders makes this less likely to be the case. As evidenced in our case report, the management of a patient with autoimmune disorders developing in association with a thymoma can become complex. Moreover, in a patient with a diagnosis of thymoma, continued surveillance is required not only for recurrence of malignant disease, but also for the development of autoimmune disorders.
